# Causal Relativistic Hydrodynamics for Viscous Fluids

**DOI:** 10.3390/e26121001

**Published:** 2024-11-21

**Authors:** Esteban Calzetta

**Affiliations:** 1 Departamento de Física, Facultad de Ciencias Exactas y Naturales, Universidad de Buenos Aires, Ciudad de Buenos Aires CP 1428, Argentina; calzetta@df.uba.ar; 2 Instituto de Física de Buenos Aires (IFIBA), CONICET—Universidad de Buenos Aires, Ciudad de Buenos Aires CP 1428, Argentina

Relativistic viscous hydrodynamics [1] was born in the works of Eckart [2] and Landau and Lifshitz [3] as a straightforward extension of the nonrelativistic Navier–Stokes equations. It faced a crisis when it became clear that this strategy led to theories with significant causality and stability issues [4]. It gained hugely in relevance when it was realized that the droplet of hadronic matter created in relativistic heavy ion collisions behaved as a viscous fluid [5,6]. It has since achieved a degree of maturity. The contributions to this Special Issue reflect its present state.

The review article by G. Rocha, D. Wagner, G. Denicol, J. Noronha, and D. Rischke [7] (see also [8]) is a critical assessment of the main issues confronting the theory and the strategies which are being pursued to deal with them. The other four contributions deal with different problems currently under research. The paper by A. Yahalom [9] deals with the Lagrangian formulation of relativistic hydrodynamics. The contribution from L. Gavassino [10] explores the range of validity of the Israel–Stewart formalism [11], taking heat conduction as leading case. The article from N. Mirón Granese, A. Kandus, and EC [12] is a detailed account of how to apply nonequilibrium field theoretic techniques [13] to describe statistical and turbulent fluctuations in relativistic fluids. Last but not least, the contribution from M. Carrington, G. Kunstatter, C. Phillips, and M. Rubio [14] studies the numerical implementation of relativistic hydrodynamics, a necessary step in the study of any real physical problem (see also [15]).

These research areas are still being vigorously pursued, and many breakthroughs have been achieved in the year or so since the publication of the last article in this Special Issue. While heavy ion collisions are still the most important application of the formalism [16,17], there are also relevant applications in cosmology [18,19,20]. At the formal level, lively research areas are establishing similarities and differences among the many formalisms being proposed [21,22,23,24], exploring new hydrodynamic theories emerging from generalized kinetic equations [25,26,27,28,29,30,31,32,33], the further clarification of the links between stability and causality [34,35,36], fluctuating relativistic hydrodynamics [37,38], and hydrodynamic models going beyond the Eckart and Landau Lifshitz frame choices [39,40,41].

One subject which is conspicuously missing from this enumeration is turbulence in relativistic fluids [42,43,44,45]. It would seem the existing formalisms for relativistic viscous fluids are too complex to be carried through to the full nonlinear regime. Yet we know that turbulence has played a major role in the dynamics of the primordial plasma in the early stages of cosmic evolution, particularly during reheating after inflation [46,47,48]. We look forward to the breakthroughs which surely are forthcoming on this important problem.
